# Construction of Baculovirus-Inducible CRISPR/Cas9 Antiviral System Targeting BmNPV in *Bombyx mori*

**DOI:** 10.3390/v14010059

**Published:** 2021-12-30

**Authors:** Yujia Liu, Dongbin Chen, Xiaoqian Zhang, Shuqing Chen, Dehong Yang, Linmeng Tang, Xu Yang, Yaohui Wang, Xingyu Luo, Manli Wang, Zhihong Hu, Yongping Huang

**Affiliations:** 1 Key Laboratory of Insect Developmental and Evolutionary Biology, Center for Excellence in Molecular Plant Sciences, Shanghai Institute of Plant Physiology and Ecology, Chinese Academy of Sciences, Shanghai 200032, China; liuyujia@cemps.ac.cn (Y.L.); sqchen@sibs.ac.cn (S.C.); yangdehong@cemps.ac.cn (D.Y.); lmtang2016@cemps.ac.cn (L.T.); yangxu@cemps.ac.cn (X.Y.); yhwang@cemps.ac.cn (Y.W.); xy384@outlook.com (X.L.); 2 University of Chinese Academy of Sciences, Beijing 100049, China; 3 Department of Sericulture, College of Bioscience and Biotechnology, Shenyang Agricultural University, Shenyang 110866, China; chendongbin@stu.syau.edu.cn; 4 China College of Forestry, Shandong Agricultural University, Taian 271018, China; ZhangXiaoqian7@aliyun.com; 5 State Key Laboratory of Virology, Wuhan Institute of Virology, Chinese Academy of Sciences, Wuhan 430071, China; Wangml@wh.iov.cn

**Keywords:** *Bombyx mori*, BmNPV, baculovirus-inducible CRISPR/Cas9, antivirus, sericulture

## Abstract

The silkworm *Bombyx mori* is an economically important insect. The sericulture industry is seriously affected by pathogen infections. Of these pathogens, *Bombyx mori* nucleopolyhedrovirus (BmNPV) causes approximately 80% of the total economic losses due to pathogen infections. We previously constructed a BmNPV-specific CRISPR/Cas9 silkworm line with significantly enhanced resistance to BmNPV. In order to optimize the resistance properties and minimize its impact on economic traits, we constructed an inducible CRISPR/Cas9 system for use in transgenic silkworms. We used the *39k* promoter, which is induced by viral infection, to express Cas9 and the U6 promoter to express four small guide RNA targeting the genes encoding BmNPV late expression factors 1 and 3 (*lef-1* and *lef-3*, respectively), which are essential for viral DNA replication. The system was rapidly activated when the silkworm was infected and showed considerably higher resistance to BmNPV infection than the wild-type silkworm. The inducible system significantly reduced the development effects due to the constitutive expression of *Cas9*. No obvious differences in developmental processes or economically important characteristics were observed between the resulting transgenic silkworms and wild-type silkworms. Adoption of this accurate and highly efficient inducible CRISPR/Cas9 system targeting BmNPV DNA replication will result in enhanced antivirus measures during sericulture, and our work also provides insights into the broader application of the CRISPR/Cas9 system in the control of infectious diseases and insect pests.

## 1. Introduction

The silkworm *Bombyx mori* is the foundation of the silk industry [1]. Silk production is impacted by biotic stresses from microbial pathogens. Infections with *B. mori* nucleopolyhedrovirus (BmNPV) cause more than 80% of the total economic losses due to pathogen infections [2]. BmNPV is a member of the *Baculoviridae* family of insect viruses. It possesses an approximately 130-kilobase, circular, double-stranded DNA genome with about 140 open reading frames [3]. Baculoviruses have a bi-directional life cycle and produce two progeny phenotypes: One is the occlusion-derived virus, which is protected by a crystallized protein coat that facilitates oral infection of the host. The other is the budded virus that plays a critical role in virus egress from midgut cells, which causes secondary infection [4]. Six genes, *ie-1*, *lef-1* (which encodes the primase), *lef-2* (which encodes a primase associated factor), *lef-3* (which encodes a single-stranded DNA binding protein), *dnapol* (which encodes the DNA polymerase), and *p143* (which encodes a helicase) are essential for baculoviral DNA replication [5,6,7,8].

Traditional antiviral strategies, such as chemical control and resistance breeding, are time-consuming and laborious, hence an increasing number of transgenic strategies have been exploited to improve the resistance of *B. mori* to infection by BmNPV [9]. Two types of strategies have been exploited to develop BmNPV-resistant transgenic silkworms: overexpression of antiviral proteins and knockdown of genes required for BmNPV replication. Overexpression of antiviral genes, such as the endogenous antiviral genes *Bmlispase-1* or *Bm-serine protease-2* (which encode proteins found in the digestive juice of *B. mori* larvae) or *BmAtlastin-n* (which encodes a member of the dynamin protein superfamily) or the exogenous antiviral gene *hycu-ep32*, have been reported to enhance the resistance of silkworm to BmNPV [10,11,12,13]. The knockdown of the BmNPV replication-related genes, such as *ie-1*, *lef-1*, *lef-2*, *lef-3*, *lef-11*, and *gp64*, also significantly inhibit virus replication [14,15,16,17,18]. However, these approaches result in only partial viral replication inhibition; they do not able to eliminate the virus completely.

The CRISPR/Cas9 system has been widely applied in cell culture and in model animals to eliminate viruses including human immunodeficiency virus 1, human papillomavirus, hepatitis B virus, Epstein-Barr virus, and plant viruses [19,20,21,22,23,24]. In a previous study, we successfully developed an antiviral strategy using a transgenic CRISPR/Cas9 system in silkworms to direct cleavage of BmNPV genomic DNA [25,26]. CRISPR/Cas9 technology has subsequently been extensively used in silkworm antiviral research. BmNPV genes, including *ie-0*, *ie-2*, *ie-1*, *me53*, *gp64*, and *lef-11*, have been targeted using CRISPR/Cas9 to improve the resistance of silkworms to BmNPV infection [27,28,29,30].

Non-specific toxicity resulting from Cas9 expression or nuclease activity has been reported [31,32,33,34]. We previously established that the constitutive expression of Cas9 and guide RNAs system resulted in higher resistance to BmNPV infection but that the transgenic homozygotes showed a 2-day developmental delay during the larval stage relative to wild-type silkworms [25]. To mitigate this toxicity, we sought to develop a system in which Cas9 protein is inducibly expressed only upon infection.

To achieve highly specific BmNPV genome editing when the silkworm has been infected by BmNPV, we constructed a BmNPV-inducible CRISPR/Cas9 system targeting the BmNPV genome. Baculovirus gene expression follows a cascade regulation pattern in which late gene expression is regulated by early gene expression [35]. Cao et al. previously screened BmNPV promoters (*VP1054*, *P33*, *Bm21*, *Bm122*, *39k*, *P143*, and *P6.9*) and found that the *39k* promoter had the highest BmNPV-induced transcriptional activity [36]. Previous studies have shown that the baculovirus Autographa californica nucleopolyhedrovirus (AcMNPV) *39k* gene is a delayed-early gene that can be transactivated by IE-0 and IE-1 [37]. Thus, in our system, Cas9 protein expression is under the control of the BmNPV-inducible *39k* promoter and expression of the four small guide RNAs (sgRNAs) is driven by the *B. mori U6* promoter. We choose the *lef-1* and *lef-3* genes as the targets to inhibit BmNPV proliferation as these genes are essential for DNA replication. Bioassay showed that the transgenic silkworm was highly resistant to BmNPV infection, and we did not observe significant differences in growth, development, or economically important traits between transgenic and wild-type silkworms. Therefore, the BmNPV-inducible system constructed to target both BmNPV *lef-1* and *lef-3* genes successfully inhibited BmNPV replication without host toxicity. The inducible anti-BmNPV therapeutic should prove useful in the sericulture industry, and similar systems could be developed to induce resistance to other pathogens and diseases.

## 2. Materials and Methods

### 2.1. Silkworm Strains and Virus Stock

The multivoltine, non-diapausing strain silkworm Nistari strain was used in this study [38]. The larvae were reared on fresh mulberry leaves under standard conditions. The BmNPV used in all of our experiments was the Zhejiang strain (GenBank accession no. JQ991008). Newly exuviated fifth instar larvae were exposed to the virus by daubing a suspension of virus on fresh mulberry leaves. The occlusion bodies (OBs) were harvested from the infected hemolymphs before the larvae died. The number of OBs was counted using a Nageotte hemocytometer and stored at 4 ℃. The concentration of virus stock solution was 10^9^ OBs/mL. The virus stock was diluted serially to prepare inocula with concentrations of 10^7^ and 10^8^ OBs/mL.

### 2.2. Target Gene Selection and Vector Construction

We selected two genes, *lef-1* and *lef-3*, as the target genes since they are essential for viral DNA replication. The *lef-1* and the *lef-3* genes are separated by a distance of ~47 kb in the BmNPV genome. The *lef1* and *lef3* target sites were designed using online software CRISPRdirect (http://crispr.dbcls.jp) (accessed date 2 December 2021) [32], and we choose the high-quality targets which conform to G-N_19_-GG consensus and were specific within the entire genome. To avoid unintended gene disruptions in silkworms, blast searches of the potential target sequences were checked with the silkworm genome in KAIKObase (http://sgp.dna.affrc.go.jp/KAIKObase/) (accessed date 16 November 2021). Four appropriate 20-base targeting sites were chosen: *lef-1*-s1 (5′-CCGCCGCACAATTGTATAGTTAC-3′) and *lef-1*-s2 (5′-GTATTGGCCGGACGTGGACAGGG-3′) for the *lef-1* gene and *lef-3*-s1 (5′ CCAAGAAGATTAGAGAAAATTAC-3′) and *lef-3*-s2 (5′- CCGATTCGGATGACCGTTCTACC-3′) for the *lef-3* gene. The *39k* promoter was cloned from the BmNPV genome (−773–+62) using the following primers: p39k-F (5′-AAGGCTGTCCTGCTGTGTGC-3′) and p39k-R (5′-CTGGCAATTCGTTTGTGATG-3′). The *39k* promoter was cloned into the *pBac* vector to construct a virus-inducible Cas9 editing system and four sgRNA targeting expression cassettes were under the separate control of the silkworm small nuclear RNA *U6* promoter. These cassettes were constructed through a series of steps to generate *pBac*-EGFP-*P39k*-Cas9/4×gRNAs. Cloning of the *IE1*-EGFP and *U6* promoters was carried out as described previously [39]. All of the primers used for vector construction are listed in Table 1.

### 2.3. Silkworm Genetic Transformation

The plasmid containing *pBac*-EGFP-*P39k*-*Cas9*/4×gRNAs and the helper plasmids were mixed and microinjected into the silkworm G_0_ embryos as previously described [25]. The injected G_0_ embryos were incubated under standard conditions until larval hatching. The G_0_ adults were mated with wild-type (WT) moths, and the G_1_-positive individuals were identified by fluorescence microscopy (Nikon AZ100) due to expression of EGFP. The insertion loci of the transgene line were identified by inverse PCR as described previously [38], and the primers used for detection are listed in Table 1.

### 2.4. Analysis of Growth and Economic Characteristics

We investigated the growth curves of transgenic lines TG-1, TG-2, and TG-3 in comparison to IE1-Cas9 transgenic silkworms [25] and that of WT larvae; every group contained at least 200 larvae. All larvae were raised under standard conditions. The larvae (*n* = 30 per group) were weighed on the fourth day of the fifth instar. All the assays were performed three times. Thirty cocoons from the TG-1, TG-2, TG-3, and WT groups were randomly selected and the sex of each cocoon was identified. For each cocoon, total cocoon weight and pupa weight were measured, and the cocoon shell rate was calculated.

### 2.5. Viral Inoculation and Mortality Analyses

The newly exuviated third-instar larvae were starved for 12 h, then each larva was fed a 1 cm^2^ piece of fresh mulberry leaf that had been smeared with 10 µL of OB suspension; suspensions of a range of concentrations were tested (10^6^, 10^5^, 10^4^ OBs/µL). We took the time when the silkworm began to ingest the virus as 0 h. It took about 12 h for a silkworm to eat the entire virus-coated leaf. Larvae were then reared on fresh mulberry leaves, and larvae from all experimental groups were maintained under the same conditions. Mortality was recorded daily for 10 days. Each group contained 60 larvae. WT larvae were treated in parallel as controls.

### 2.6. RNA Isolation and cDNA Synthesis

Total RNA was extracted from silkworm using Trizol reagent (10606ES60, Yeasen, Shanghai, China) and treated with RNase-free DNaseI (2270A, TaKaRa, Kusatsu, Japan). The quality of total RNA was confirmed by agarose gel electrophoresis. For each sample, cDNA was synthesized from an aliquot of 1 μg of the total RNA using the ReverAid First Strand cDNA Synthesis Kit (K1622, TaKaRa, Kusatsu, Japan).

### 2.7. Time-Course qPCR Analysis of BmNPV DNA Copies after Oral Infection

The real-time virus accumulation level was determined by the measurement of viral DNA copies. Whole larvae samples were separately collected from silkworms in the TG-1, TG-2, TG-3, and WT groups treated with 10^6^ OBs/larva (*n* = 6 per group) once every 12 h (from 12 to 84 hpi). Total DNA was extracted with a Tissue DNA kit (D3396-01, OMEGA, Norcross, USA) and then treated with RNase A (2158, TaKaRa, Kusatsu, Japan). The DNA templates (60 ng) were amplified with primers that bind to the BmNPV *ie-1*, *gp64*, and *p10* genes. Amplification of *Bmrp49*, the gene encoding *B. mori* ribosomal protein 49 was used as an internal control. Quantitative PCR (qPCR) analysis was performed with an Eppendorf Mastercycler ep realplex with SYBR Green Real-Time PCR Master Mix (QPK-201C, Toyobo, Kusatsu, Japan). The amplification program used was incubation at 95 °C for 3 min, followed by 40 cycles of 95 °C for 15 s, and 60 °C for 1 min. Standard curves were determined with 5-fold serially diluted DNA. The data were analyzed using GraphPad Prism version 7. The sequences of all of the qPCR primers used are listed in Table 1. The test was performed three times.

### 2.8. Quantification of BmNPV Gene Expression

Whole larvae samples were separately collected from silkworms treated with 10^6^ OBs/larva in the TG-1, TG-2, TG-3, and WT groups (*n* = 6 per group) at 72 hpi. The expression levels of several BmNPV genes (immediate early gene *ie-1*, early gene *p143*, late gene *vp39*, and very late gene *p10*) were analyzed by quantitative real-time RT-PCR (qRT-PCR). qRT-PCR assays were performed using SYBR Green Real-time PCR Master Mix (QPK-201C, Toyobo, Kusatsu, Japan) on an Eppendorf Real-time PCR System Mastercycler realplex. A 5-fold serial dilution of pooled cDNA was used as the template to make standard curves. Quantitative mRNA measurements were performed in three independent biological replicates and normalized to *Bmrp49* mRNA.

### 2.9. Mutagenesis Analysis of the Viral Target Genes

Total DNA was extracted from six whole larvae treated with 10^6^ OBs/larvae in the TG-1 group and six whole larvae treated with 10^6^ OBs/larvae in the WT group at 24 hpi using standard SDS lysis-phenol treatment. Samples were incubated with proteinase K (B600452-0001, BBI, Shanghai, China), treated with RNase treatment, and purified. To perform gene-specific PCR amplification, 100 ng of the total DNA was used as the template. The primers were designed to detect mutagenesis at target sites. The primers *lef-1*-mut-F and *lef-1*-mut-R detected mutagenesis at target sites 1 and 2 in the *lef-1* gene. The primers *lef-3*-mut-F and *lef-3*-mut-R detected mutagenesis at targeting sites 1 and 2 in the *lef-3* gene. The PCR products were extracted and cloned into the pJET-1.2 vector, and 20 clones from five individuals were sequenced. The sequences of the primers used are listed in Table 1.

### 2.10. Statistical Analysis

All of the experiments in this study were performed with at least three replicates. All data are expressed as the mean ± standard error of the mean (SEM). The differences between groups were examined by either two-tailed Student’s *t*-test, one-way ANOVA, or two-way ANOVA. All statistical calculations were performed and graphs were made with GraphPad Prism version 7.0. Statistically significant differences are indicated by asterisks.

## 3. Results

### 3.1. Construction of Baculovirus-Inducible CRISPR/Cas9 Gene Editing System

**Figure 1:**
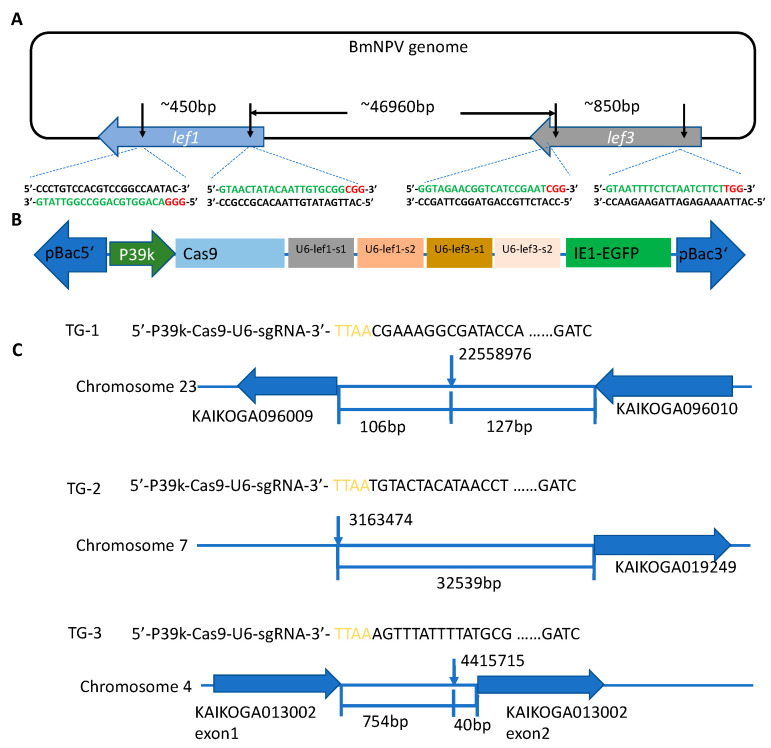
Construction of the BmNPV-inducing and targeting CRISPR/Cas9 gene-editing system in the silkworm. (**A**) Schematic representation of the locations of sequences targeted by sgRNAs in *lef-1* (blue arrow) and *lef-3* (grey arrow) in the BmNPV genome. The sgRNA target sequences are green, and the PAM sequences are red. The distances between target sites are given in base pairs. (**B**) Design of the *pBac*-EGFP-*P39k*-*Cas9*/4×gRNAs construct. The green arrow represents the *39k* promoter and the blue box represents the *cas9* coding region. The gray, blue, brown, and pink boxes represent sgRNA expression cassettes targeting *lef-1*-s1, *lef-1*-s2, *lef-3*-s1, and *lef-3*-s2, respectively. The green box represents the EGFP expression cassette. (**C**) Schematic of insertion sites of the *pBac-EGFP-P39k-Cas9*/4×gRNAs construct in the TG-1, TG-2, and TG-3 lines. The vertical arrows indicate the insertion sites. The horizontal blue arrows represent genes adjacent to the insertion sites in TG-1 and TG-2 and the gene disrupted in TG-3. The distances are given in base pairs.

The expression of baculovirus follows a cascade that requires early expression proteins to trans-activate late gene expression. Previous studies have shown that the baculovirus IE-1 protein binds to the *39k* promoter and activates the expression of the late protein 39K [40]. Based on this, we constructed a baculovirus-inducible CRISPR/Cas9 gene-editing system for use in silkworms. First, we cloned the *39k* promoter from the BmNPV genome (Zhejiang strain) to control the expression of *Cas9*. Then we selected the *lef-1* and *lef-3* genes of BmNPV as targets for sgRNAs. The two genes are separated by about 47 kilobases in the BmNPV genome (Figure 1A). Three different expression cassettes were integrated into the piggyBac-based transgenic plasmid, *pBac*-EGFP-*P39k*-*Cas9*/4×gRNAs (Figure 1B). Eight healthy transgenic lines were obtained and we selected three (TG-1, TG-2, and TG-3) of them for sequence analyses. The cassettes did not insert into exons of critical genes. The insertion sites in TG-1 and TG-2 were not in intronic or exonic regions, whereas in TG-3 the insertion was in the first intron of a gene with unknown function (Figure 1C).

### 3.2. TG Silkworms Exhibit Higher Resistance to BmNPV Infection Than the Wild-Type Silkworms

**Figure 2:**
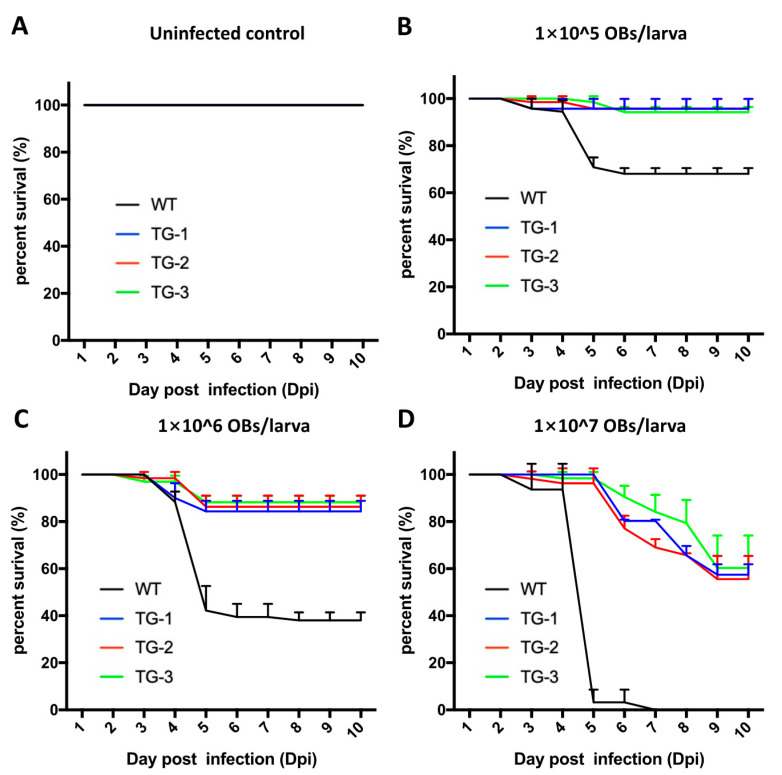
Resistance to BmNPV infection in transgenic and wild-type silkworms. (**A**) Survival rate analysis of TG and WT silkworms without treatment with BmNPV. (**B**–**D**) Survival rate analysis of TG and WT silkworms after inoculation with (**B**) 1 × 10^5^ OBs/larva, (**C**) 1 × 10^6^ OBs/larva, and (**D**) 1 × 10^7^ OBs/larva. Values are the means of three independent replicates with 30 larvae in each replicate. Statistical analysis of mortality was conducted 10 days after inoculation with OBs.

We then tested the resistance of wild-type and transgenic silkworm lines to infection with BmNPV by counting the number of silkworms that survived each day for 10 days post infection (dpi). All of the individuals in the uninfected control groups survived throughout the experiment (Figure 2A; *n* = 60 for every line). At a dose of 10^5^ OBs/larva, the survival rate of transgenic lines was approximately 94% at 10 dpi, but the survival rate of WT silkworms was only 68% (Figure 2B). After inoculation with 10^6^ OBs/larva, the average survival rate of TG-1, TG-2, and TG-3 silkworms were 84%, 86%, and 88%, respectively. In contrast, the survival rate of WT larvae was only 38% (Figure 2C). All WT silkworms were dead at 6 dpi after inoculation with 10^7^ OBs/larva, but the survival rate of transgenic silkworms remained above 50% through 10 dpi (Figure 2D). These results demonstrated that the BmNPV-inducible CRISPR/Cas9 system that expresses sgRNAs targeting *lef-1* and *lef-3* upon BmNPV infection dramatically improved silkworm survival.

### 3.3. CRISPR/Cas9-Mediated BmNPV Genome Editing in Transgenic Silkworm

**Figure 3:**
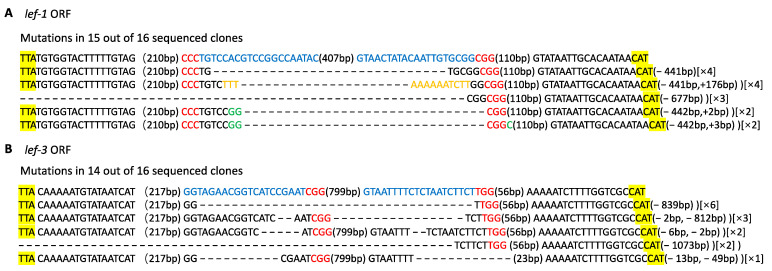
CRISPR/Cas9-mediated BmNPV genome editing in transgenic silkworms. Mutations detected in (**A**) *lef-1* and (**B**) *lef-3* in TG-1 silkworms inoculated with 10^6^ OBs/larva. For each gene, the open reading frame in the WT sequence is shown at the top with the start and stop codons highlighted in yellow, the target sites in blue, and the PAM sequences in red. The net change in length caused by each indel mutation is given to the right of the sequence (+, insertion; −, deletion). The number of times each mutant clone was isolated is shown in brackets.

**Figure 4:**
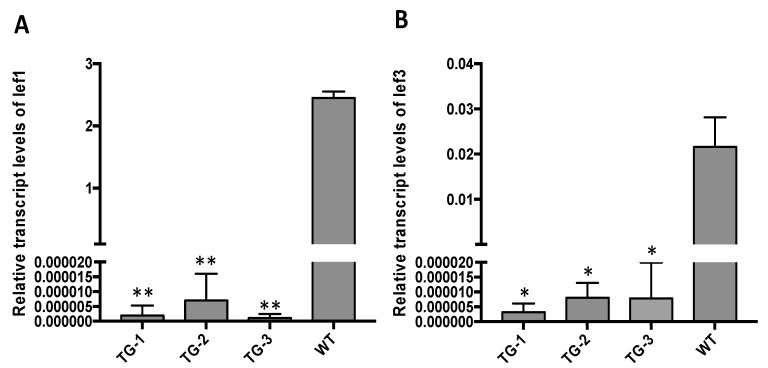
Expression levels of BmNPV *lef-1* and *lef-3* mRNAs in transgenic and wild-type silkworms treated with 10^6^ OBs/larva at 72 hpi. The relative transcript levels of (**A**) *lef-1* and (**B**) *lef-3* are shown. Data represent the mean ± SEM. * *p* < 0.05, ** *p* < 0.01, by one-way ANOVA.

We compared the viral sequences of different BmNPV strains and found that the sequences targeted in *lef-1* and *lef-3* were conserved among different BmNPV genotypes. In order to confirm that the two target genes were successfully disrupted by the CRISPR/Cas9 system, we sequenced *lef-1* and *lef-3* of BmNPV isolated from TG-1 silkworms infected with 10^6^ OBs/larva. Total DNA from TG-1 and WT larva were isolated at 24 hpi and relevant regions of *lef-1* and *lef-3* were sequenced. Many types of mutations were detected in these genes collected from TG-1 silkworms including insertions and small and large deletions (Figure 3). In the clones isolated from WT silkworms, no gene editing was detected in the BmNPV genes. The most frequent mutation detected in the virus isolated from the transgenic silkworms was a small-segment deletion of the sequence from the PAM of *lef-1*-s1 to the PAM of *lef-1*-s2 in the open reading frame of the *lef-1* gene. In addition, insertions were detected between the two target sites. Sequencing of the open reading frame of *lef-3* gene from the infected transgenic larvae identified deletion events and small-segment deletion events. We also quantified *lef-1* and *lef-3* mRNAs. These mRNAs were almost undetectable in the transgenic animals infected with the virus but were highly expressed in WT silkworms (Figure 4). We also tried to amplify the region spanning *lef-1* and *lef-3*, but no significant products were found (data not shown). The *lef-1* and *lef-3* genes are separated by a distance of ~47 kb in the BmNPV genome, which is likely too far apart to be completely removed.

### 3.4. The Antivirus Efficiency of the Baculovirus-Inducing and Targeting CRISPR/Cas9 System Is High

**Figure 5:**
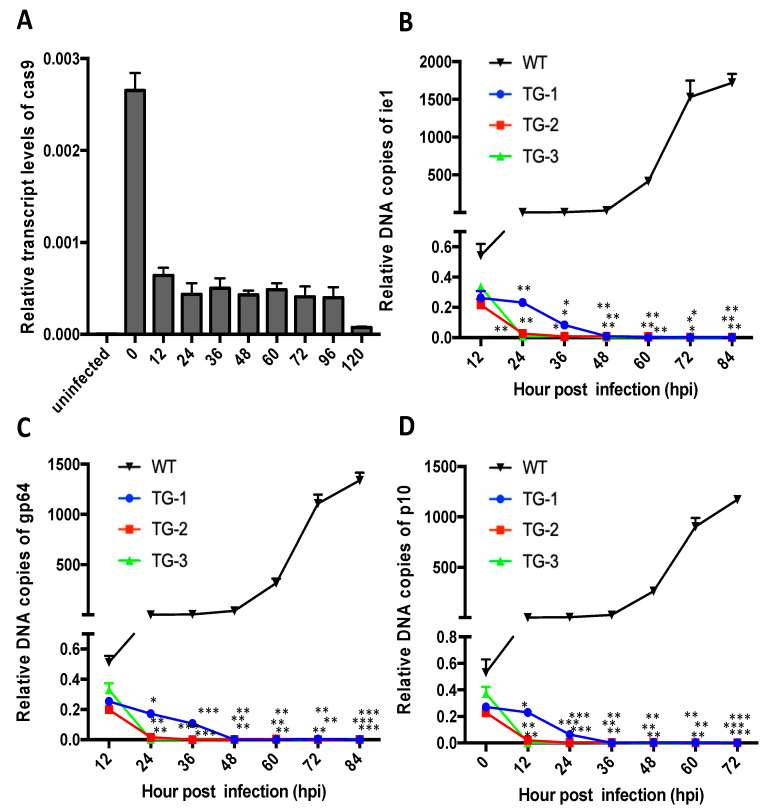
Antiviral efficiency of the BmNPV-inducible CRISPR/Cas9 system. (**A**) qRT-PCR analysis of *Cas9* mRNA in TG-1 silkworms infected with 10^6^ OBs/larva. (**B**–**D**) Relative numbers of copies of BmNPV genes (**B**) *ie-1*, (**C**) *gp64*, and (**D**) *p10* determined by qPCR at different time points after viral inoculation with 10^6^ OBs/larva. Data represent the means ± SEM. * *p* < 0.05; ** *p*< 0.01; *** *p* < 0.001 by two-way ANOVA.

To more accurately evaluate the antiviral efficiency of the TG silkworm lines, we investigated the relative number of DNA copies of BmNPV genes to monitor viral proliferation in TG and WT silkworms. The genes *ie-1*, *gp64*, and *p10*, which are present in single copies in the BmNPV genome, were selected as indicators in a qPCR analysis for relative copies of BmNPV. We first determined the sensitivity of the BmNPV-inducible CRISPR/Cas9 system to BmNPV infection by evaluating the expression pattern of *cas9* after inoculation of TG-1 silkworms with 1×10^6^ OBs/larva over a time course from 0 to 84 h post infection (hpi). The BmNPV-inducible CRISPR/Cas9 system was activated rapidly with a peak at 12 hpi (Figure 5A). Consistent with *cas9* expression, the relative numbers of copies of *ie-1*, *gp64*, and *p10* were the highest at 12 hpi in the transgenic animals, and then significantly decreased over time. At 84 h post infection in transgenic silkworms, the viral DNA was barely detected, and the expression of cas9 was significantly decreased (Figure 5B–D). In contrast, the viral DNA copies in WT silkworms increased throughout the time course and had not plateaued at 84 hpi (Figure 5B–D).

**Figure 6:**
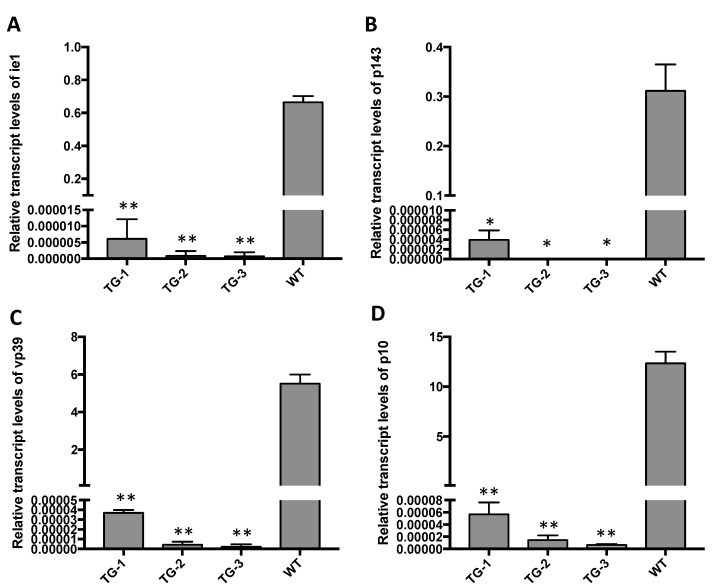
Expression levels of different-phase BmNPV genes in transgenic and WT silkworms. The relative transcript levels of (**A**) *ie-1*, (**B**) *p143*, (**C**) *vp39*, and (**D**) *p10* were determined by qRT-PCR in transgenic larvae and wildtype larvae treated with 10^6^ OBs/larva at 72 hpi. Data represent the means ± SEM. * *p* < 0.05; ** *p* < 0.01 by one-way ANOVA.

To further evaluate inhibition of BmNPV gene expression, total RNA was extracted from the TG silkworms and WT silkworms at 72 hpi, and the expression levels of several BmNPV mRNAs were analyzed by qRT-PCR. BmNPV *ie-1*, *p143*, *vp39*, and *p10* were expressed at very low levels in the transgenic larvae after inoculation with 1×10^6^ OBs/larva (Figure 6). In contrast, the expressions of these mRNAs were significantly higher in WT silkworms.

### 3.5. Economically Important Characteristics Are Normal in the Transgenic Silkworm

**Figure 7:**
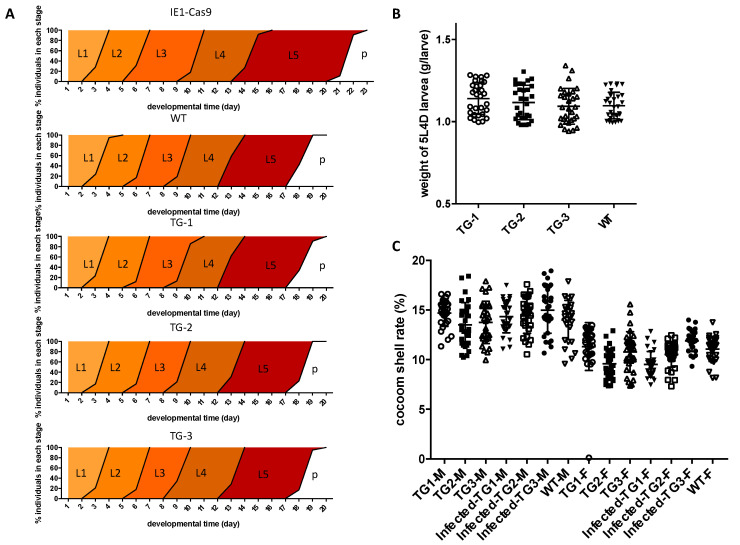
Economically important characteristics transgenic silkworms. (**A**) The timing of larval development in the virus-inducible transgenic, IE1-Cas9 transgenic, and WT silkworms (L1, first instar; L2, second instar; L3, third instar; L4, fourth instar; L5, fifth instar; *p*, pupae). (**B**) Body weights of 5L4D transgenic silkworm larvae and WT larvae (200 larvae from each group were evaluated). (**C**) Cocoon shell rate of the transgenic and WT lines. The infected TGs were the transgenic silkworms that survived after inoculation with 1 × 10^6^ OBs/larva. Each value represents the average of 30 larvae per group (M, male; F, female).

To determine the impact of the virus-inducible CRISPR/Cas9 system on silkworm development, we analyzed the growth and development of different transgenic silkworm larvae, IE1-Cas9 homozygotes, and wildtype larvae at the same time. We found no significant differences in developmental progression between the virus-inducible transgenic and WT lines; in contrast, the IE1-Cas9 homozygotes that we constructed previously [25] showed a 2-day developmental delay during the larval stages (Figure 7A). There were no significant differences in larval weight in L5D4 between the virus-inducible transgenic silkworms and WT silkworms (Figure 7B). The transgenic silkworms that survived after inoculation with 1 × 10^6^ OBs/larva also had shell rates that were not significantly different from those of uninfected WT silkworms (Figure 7C). Thus, the BmNPV-inducible Cas9 system effectively edited the target gene without any significant effects on larval development or economically important characteristics.

## 4. Discussion

The silkworm is an economically important insect, and the disease caused by BmNPV results in huge economic losses in the sericulture industry [41]. We previously established a transgenic CRISPR/Cas9 system in silkworms and showed that the transgenic animals had higher resistance to BmNPV infection than the WT animals and that the resistance was inheritable [25]. Subsequently, the viral genes, *ie-1*, *ie-0*, and *ie-2*, were targeted to construct antiviral transgenic silkworms [27,30]. Recently, the ATPase family AAA domain-containing protein 3 (ATAD3A) and the BmNPV inhibitor of apoptosis protein 2 (*iap2*) have been targeted using a CRISPR/Cas9 system to engineer silkworms resistant to BmNPV [28,42]. There is a certain risk to knock out endogenous genes of silkworms, which may affect the growth and development of silkworm or the expression of adjacent genes in the silkworm genome. The use of the CRISPR system to impart resistance to insect pathogens has not been widely implemented in part due to adverse impacts of constitutive Cas9 expression. In silkworms, the constitutive expression of Cas9 can result in changes in larval development. Antiviral strategies must balance disease resistance and economic characteristics [2]. The BmNPV-inducible transgenic CRISPR/Cas9 system reported here imparted dramatic resistance to BmNPV without economically important characteristics. The baculovirus-inducible CRISPR/Cas9 system we developed targets critical BmNPV genes *lef-1* and *lef-3*. Only upon viral infection are Cas9 and sgRNA induced, and the system successfully disrupted BmNPV replication.

Whereas the homozygotes constitutively expressing *cas9* showed a 2-day developmental delay during the larval stage compared to WT, the virus-inducible transgenic lines constructed for this study showed no significant differences in developmental progression or larval weight compared to WT. The BmNPV-inducible CRISPR/Cas9 system was rapidly activated when the silkworm was infected with BmNPV, and when virus was cleared, the expression of *cas9* decreased significantly. There were no significant differences in cocoon shell rates between transgenic silkworms, even those that survived BmNPV infection, and the WT silkworms.

The genes we targeted, *lef-1* and the *lef-3*, are essential for BmNPV DNA replication and had not previously been targeted to construct CRISPR/Cas9 anti-virus transgenic silkworms. We designed sgRNAs to two target sites in each gene, and the qPCR showed that both were successfully knocked out in the transgenic larvae infected with BmNPV. This system could be used to target other genes in BmNPV or genes expressed by other pathogenic microorganisms such as bacteria or fungi that infect silkworms or other viruses such as *B. mori* densovirus. Screening endogenous or exogenous genes that are expressed after pathogenic invasion and adapting the promoter sequences of these genes to express proteins that impart resistance will provide insights into silkworm gene function and disease resistance.

In addition to the CRISPR/Cas9 technology, the CRISPR/Cpf1 system has been used to achieve high editing efficiencies in the silkworm [43]. The CRISPR/Cas13 system, which is the only known system that can target RNA for cleavage, could be used to engineer silkworm resistant to *B. mori* cypovirus, a virus with an RNA genome [44,45,46,47].

In conclusion, we established a highly efficient BmNPV-inducible CRISPR/Cas9 system that targets two essential genes of BmNPV. There were no obvious differences in developmental processes or economic characteristics between the transgenic silkworms and the wild-type silkworm. Thus, the use of a virus-inducible system resulted in silkworms being resistant to a virus that causes considerable economic losses in the silkworm industry without host toxicity.

## Figures and Tables

**Table 1:** Primers used in this study.

Primer Name	Primer Sequence (5′-3′)	Primer Purpose
*P39k*-F	AAGGCTGTCCTGCTGTGTGC	Plasmid construction
*P39k*-R	CTGGCAATTCGTTTGTGATG	Plasmid construction
*P39k*-cas9-F	ACTCACTATAGGGCGAATTGGGTACCGTCGACAAGGCTG TCCTGCTGTGTGC	Plasmid construction
*P39k*-cas9-R	ATGGAGTACTTCTTGTCCATGACGTCCTGGCAATTCGTTTGTGATG	Plasmid construction
*lef-1*-sg1-F	GTAACTATACAATTGTGCGGGTTTTAGAGCTAGAAATAGC	Plasmid construction
*lef-1*-sg1-R	CCGCACAATTGTATAGTTACACTTGTAGAGCACGATATTT	Plasmid construction
*lef-1*-sg2-F	GTATTGGCCGGACGTGGACAGTTTTAGAGCTAGAAATAGC	Plasmid construction
*lef-1*-sg2-R	TGTCCACGTCCGGCCAATACACTTGTAGAGCACGATATTT	Plasmid construction
*lef-3*-sg1-F	GTAATTTTCTCTAATCTTCTGTTTTAGAGCTAGAAATAGC	Plasmid construction
*lef-3*-g1-R	AGAAGATTAGAGAAAATTACACTTGTAGAGCACGATATTT	Plasmid construction
*lef-3*-g2-F	GGTAGAACGGTCATCCGAATGTTTTAGAGCTAGAAATAGC	Plasmid construction
*lef-3*-g2-R	ATTCGGATGACCGTTCTACCACTTGTAGAGCACGATATTT	Plasmid construction
*U6*-F1	ACTCACTATAGGGCGAATTGGGTACCAGGTTATGTAGTACACATTG	Plasmid construction
Overlap-gBone-R	CCGCGGAGTCAATGGCTAGCAAAAAAAGCACCGACTCGGTG	Plasmid construction
Overlap-*U6*-F	GCTAGCCATTGACTCCGCGGAGGTTATGTAGTACACATTGTTGTA	Plasmid construction
gBone-HindIII-R	TTTTCTTGTTATAGATATCAAAAAAAAGCACCGACTCGGTG	Plasmid construction
*ie1*-qPCR-F	GCTCAAGACCACTGATAATCTC	qPCR
*ie1*-qPCR-R	AATCGTCCAAGTATTCGTCCA	qPCR
*gp64*-qPCR-F	CCTTCAGCCATGGAAGTGAT	qPCR
*gp64*-qPCR-R	GACGACGTCGAATTTTGGAT	qPCR
*p10*-qPCR-F	CCATTGCGGAAACTAACACA	qPCR
*p10*-qPCR-R	AGCAGTGTCACCGGTCAATA	qPCR
*Bmrp49*-qPCR-F	AAACATACAAGATGGCTATAAGACCTG	qPCR
*Bmrp49*-qPCR-R	TTTATAAATGACATGTGAACATACCTC	qPCR
Inverse PCR-F1	CAGTGACACTTACCGCATTGA	Inverse PCR
Inverse PCR-R1	CATTTTGACTCACGCGGTC	Inverse PCR
Inverse PCR-F2	CGCTATTTAGAAAGAGAGAGCAA	Inverse PCR
Inverse PCR-R2	ATCACGTAAGTAGAACATGAAATAACA	Inverse PCR
*ie1*-qRT-F	GCTCAAGACCACTGATAATCTC	qPCR
*ie1*-qRT-R	AATCGTCCAAGTATTCGTCCA	qPCR
*p143*-qRT-F	TGGCTTCATACTTTAGCAACC	qPCR
*p143*-qRT-R	GTTTGACGATGACAACCACAG	qPCR
*vp39*-qRT-F	TCTAAATCTCAATTCCTCCGTG	qPCR
*vp39*-qRT-R	GCATTCTAGACACCACAAACC	qPCR
*p10*-qRT-F	CCATTGCGGAAACTAACACA	qPCR
*p10*-qRT-R	AGCAGTGTCACCGGTCAATA	qPCR
*Bmrp49*-qRT-F	TCAATCGGATCGCTATGACA	qPCR
*Bmrp49*-qRT-R	ATGACGGGTCTTCTTGTTGG	qPCR
*lef-1*-mut-F1	GCGCCCAACGAGTTGAGATC	Mutagenesis detection
*lef-1*-mut-F2	GCAATCAAGTATGTCGTCGT	Mutagenesis detection
*lef-1*-mut-R1	AACTTCCGTAATATTACCGC	Mutagenesis detection
*lef-1*-mut-R2	TTGGGCTTGACGACCGTAAC	Mutagenesis detection
*lef-3*-mut-F1	GACGAGCGATTCCAAAACTT	Mutagenesis detection
*lef-3*-mut-F2	GCAGATCAGGCTGTCAAATC	Mutagenesis detection
*lef-3*-mut-R1	GCTTCGTGTCGGTCGTACGG	Mutagenesis detection
*lef-3*-mut-R2	ATCGTTAAATCGAGCGGGTC	Mutagenesis detection
